# Autopsy diagnosis of acupuncture-induced bilateral tension pneumothorax using whole-body postmortem computed tomography: A case report

**DOI:** 10.1097/MD.0000000000013059

**Published:** 2018-11-02

**Authors:** Junqi Jian, Yu Shao, Lei Wan, Min Zhang, Ningguo Liu, Jianhua Zhang, Yijiu Chen

**Affiliations:** a From the Shanghai Key Laboratory of Forensic Medicine, Shanghai Forensic Service Platform, Academy of Forensic Science, Shanghai; b Affiliated Hospital of Nantong University, Jiangsu, China.

**Keywords:** acupuncture, pneumothorax, postmortem computed tomography (PMCT), postmortem forensic imaging

## Abstract

**Rationale:**

Acupuncture, a component of traditional Chinese medicine, is also a well-known form of complementary and alternative medicine. Serious adverse events of acupuncture have been reported, including the acupuncture-related pneumothorax which is a rare but fatal condition sometimes. The pneumothorax was related to needle insertion in the upper back or paraspinal area and the reported victims suffered from either unilateral or bilateral pneumothorax. Postmortem computed tomography has advantages in the detection of pathologic gas and is being considered as a useful visualization tool for diagnosing the cause of death.

**Patient concerns:**

A 52-year-old man underwent acupuncture and cupping treatment at an illegal Chinese medicine clinic for neck and back discomfort and was admitted to the hospital with severe gasp and dyspnea about 30 hours later. The patient suddenly became unconscious with heart rate and blood pressure lost and died after cardiopulmonary resuscitation. Diagnosis, interventions and outcomes: Whole-body postmortem computed tomography of the victim revealed collapse of the both lungs and mediastinal compression, which was also confirmed by autopsy. More than 20 pinprick injuries were found on the skin of the upper and lower back in which multiple pinpricks were located on the body surface projection of the lungs. The cause of death was concluded as acute respiratory and circulatory failure due to acupuncture-induced bilateral tension pneumothorax.

**Lessons:**

Acupuncture-induced tension pneumothorax is rare and should be recognized by forensic pathologists. Postmortem computed tomography can be used to detect and accurately evaluate the severity of pneumothorax before autopsy and can play a supporting role in determining the cause of death.

## 1 Introduction

Acupuncture, a component of traditional Chinese medicine, is also a well-known form of complementary and alternative medicine.^[1,2]^ However, the true effect of acupuncture is still under debate. There is evidence for its efficacy in the treatment of chronic pain conditions such as neck and back pain, osteoarthritis, and chronic headache and shoulder pain.^[1]^ Other reports consider acupuncture as a form of integrative medicine that works solely through the placebo effect.^[3,4]^ However, serious adverse events (AEs) of acupuncture have also been reported.^[5–7]^ Since the occurrence of most cases of acupuncture-related pneumothorax is delayed, this complication is usually under recognized by acupuncturists and mainly addressed by hospital and emergency room physicians.

In recent years, postmortem computed tomography (PMCT) is being considered as a useful visualization tool for diagnosing the cause of death during forensic practices.^[8,9]^ It has prominent advantages in the detection of both pathologic gas collections and fractures.^[10]^ At autopsy, the pneumothorax test may be ignored as a nonroutine dissection procedure. Furthermore, it is difficult to detect the presence of gas in the pleural space after exposing the chest cavity to the external environment. Bilateral pneumothorax will lead to the collapse of both lungs, while unilateral pneumothorax causes a shift in the mediastinum toward the intact side. In this case, critical information received from PMCT imaging helped forensic pathologist to easily determine the cause of death before the conventional autopsy.

## 2 Case report

A 52-year-old man underwent acupuncture and cupping treatment at an illegal Chinese medicine clinic for neck and back discomfort. Multiple 0.25 mm × 75 mm needles were utilized and the acupuncture points were located in the middle and on both sides of the upper back and the middle of the lower back. The acupuncture and subsequent cupping treatment lasted 30 minutes, respectively. The patient presented to the hospital with severe gasp and dyspnea about 30 hours later. Physical examinations were as follows: blood pressure (BP) was 149/94 mm Hg, heart rate (HR) was 86 beats/min, and blood oxygen saturation level was 54%. The patient was lucid, was gasping, and had apnea and low respiratory murmur, accompanied by some wheeze in both sides of the lungs. Because of the respiratory difficulty, the patient could hardly speak. After primary physical examination, he was suspected of having foreign body airway obstruction. Around 30 minutes after admission, the patient suddenly became unconscious with HR and BP not being measured. The patient died after an hour of cardiopulmonary resuscitation.

This study was approved by the Academic Committee of the Institute of Forensic Science, Ministry of Justice, People's Republic of China. Written informed consents were obtained from the victim's family to publish these case details.

## 3 Postmortem computed tomography (PMCT)

The time interval between death and PMCT scanning was about 207 hours. The corpse was frozen after death and thawed before examination. The whole body was scanned using a 40-slice multislice CT system (Definition AS; Siemens Healthineers, Erlangen, Germany). Raw data were acquired using the following settings: voltage 120 kV, current 240 mAs, collimation 6.0 × 1.0 mm. Image reconstruction was achieved at slice thicknesses of 5.0 and 0.625 mm, each with an increment of half the slice thickness (soft tissue and bone-weighted reconstruction kernel). Image review and three-dimensional (3D) reconstructions were performed using Mimics 14.0 software (Materialise Inc., Leuven, Belgium).

## 4 Autopsy and other analysis

Conventional autopsy was performed about one hour after the PMCT examination. During the autopsy, external and internal examinations of the body were performed. Histological samples of most of the organs within the cranial, thoracic, and abdominal cavities were subjected to hematoxylin & eosin (H&E) staining. Blood was sent for toxicological analyses.

## 5 Postmortem computed tomography (PMCT) findings

**Figure 1:**
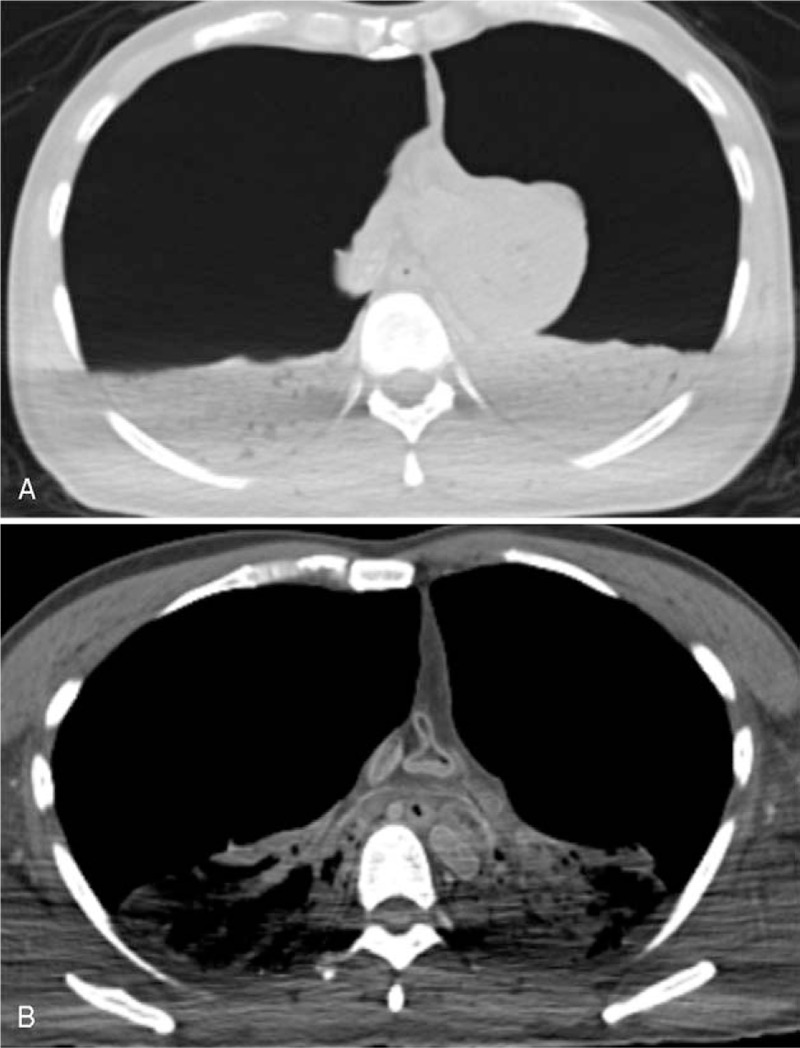
PMCT findings in (A) the lung window, (B) the mediastinal window. Both lungs were collapsed and the density of both lungs was significantly elevated. Mediastinal compression and compression of heart and major vessels were also detected. PMCT = postmortem computed tomography.

**Figure 2:**
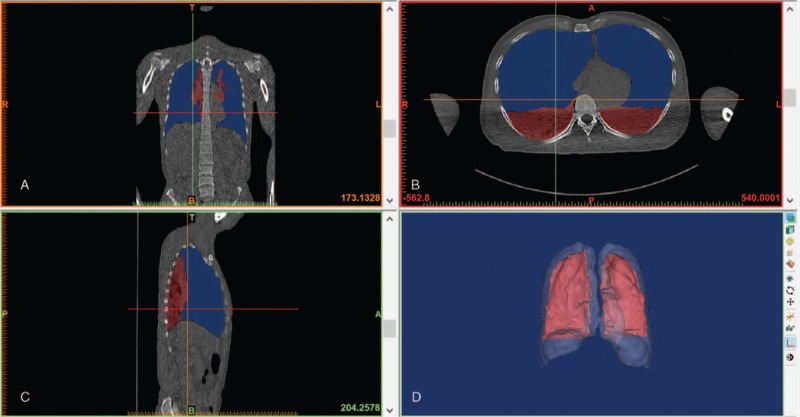
Screen images of 3D reconstructions of lungs. Views in the coronal (A), axial (B) and sagittal (C) planes showed the collapsed lungs (purple) and gas in the pleural space (blue). 3D reconstructions (D) showed the collapsed lungs (red) and gas in pleural space (translucent).

PMCT results showed that both lungs had collapsed and the density of both lungs was significantly elevated (Fig. 1). Mediastinal compression and compression of the heart and major vessels were also detected. 3D reconstruction results revealed that the both lungs had collapsed to about 20% of their original size (Fig. 2).

## 6 Autopsy, histological findings, and toxicological analysis

**Figure 3:**
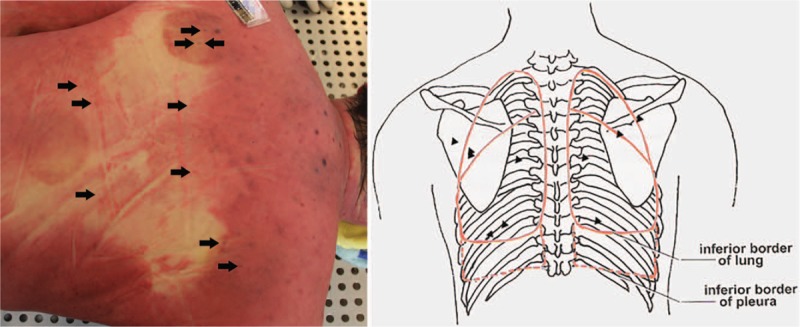
Autopsy findings. Multiple acupuncture injuries (arrows) and cupping marks were found in the upper back. The location of the acupuncture points are illustrated with black triangles.

**Figure 4:**
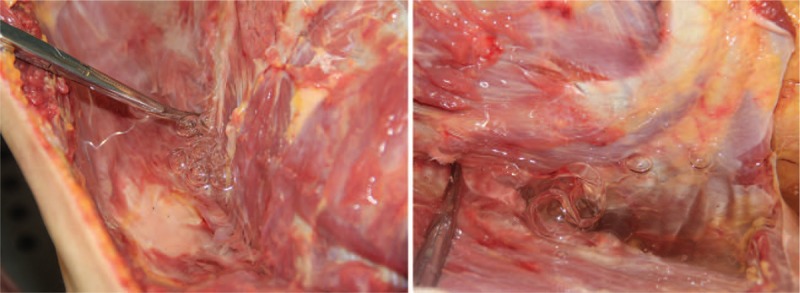
Autopsy findings. Results of bilateral pneumothorax test.

**Figure 5:**
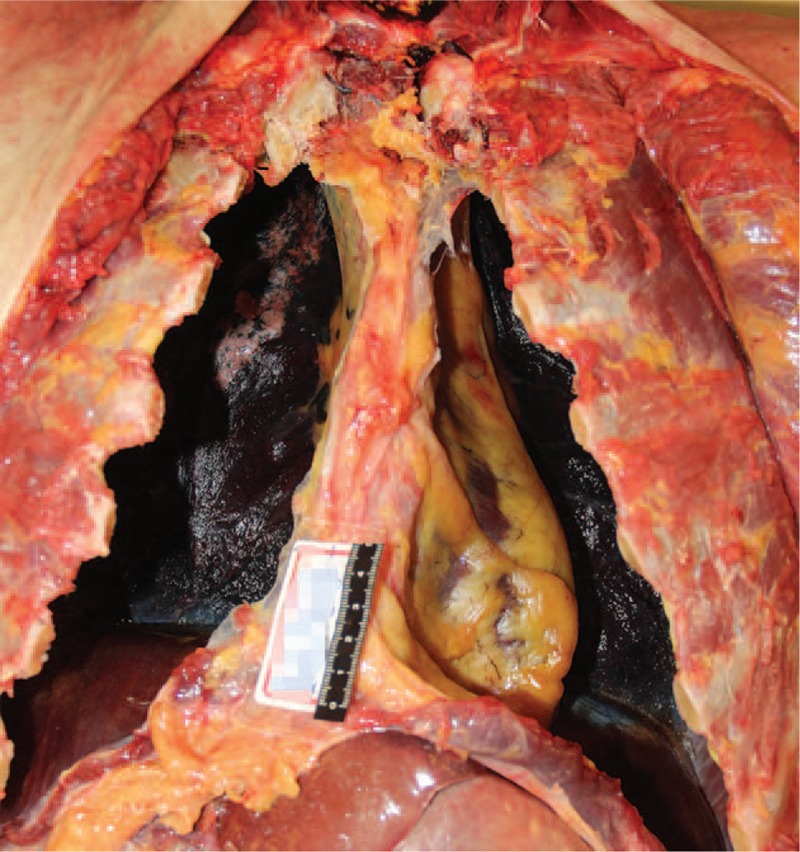
Autopsy findings. Both lungs were greatly collapsed to the mediastinum and posterior thoracic cavity wall. Compression and dextroposition of heart were also noted.

External examination revealed that the deceased was of medium body shape and without developmental malformations. Multiple quasi-circular marks due to cupping were found on the both sides of the upper back and the middle of the lower back. In total, more than 20 pinprick injuries from the acupuncture were found on the skin of the upper and lower back. Five pinpricks were found above the scapulae, with 3 on the left, and 2 on the right side, respectively. Two pinpricks were located approximately lateral to the sixth thoracic vertebra on both sides, in the region between the scapular margin and thoracic spine. Two pinpricks were found on the left side, and one pinprick on the right side of the upper back, approximately lateral to the tenth thoracic vertebra, below the scapular margin. More than ten pinpricks were found on the lower back (Fig. 3). No other injuries were revealed. During internal examination, bilateral pneumothorax tests were performed by standard means, the results of which were positive (Fig. 4). Both lungs were greatly collapsed toward the mediastinum and posterior thoracic cavity wall (Fig. 5). The left lung weighed 512 g, and the right lung weighed 581 g. The surface of the lungs was smooth with multiple dust-like black spots on the visceral pleura. No significant injury or hemorrhagic spots were found in either the parietal or visceral pleura. Compression and dextroposition of heart was detected, which was located close to the mediastinum. The weight of the heart was 328 g. The epicardium and endocardium were smooth and without hemorrhagic spots. No hydrothoraxes were detected, nor were any other abnormalities found.

Histological examination revealed local atelectasis, blood congestion of alveolar capillaries, as well as pulmonary oedema and local emphysema. Dust-like black pigments were also discovered beneath the visceral pleura. Blood congestion of other organs was also found, however, no other abnormalities were found. Toxicological results were negative for alcohol and common drugs.

The cause of death was concluded as acute respiratory and circulatory failure due to bilateral tension pneumothorax, which was caused by acupuncture in the paraspinal areas on both sides of the upper back.

## 7 Discussion

Pneumothorax refers to gas accumulation in the pleural space and is considered a rare phenomenon compared to other severe AEs of acupuncture, such as central nervous system injury, infection, epidural hematoma, subarachnoid hemorrhage, cardiac tamponade, gallbladder perforation, and hepatitis.^[11]^ Pneumothorax could be spontaneous, traumatic, or iatrogenic. During acupuncture, especially when performed by untrained personnel, the needle may be inserted into the pleural space or even into the lungs themselves, leading to tension pneumothorax. In this case, the pathophysiology of the bilateral pneumothorax is most likely as follows: perforation of the lungs occurred when the acupuncture needles were inserted into the thoracic cavities from the paraspinal areas in both sides of the upper back. Five pinpricks in the back were located on the body surface projection of the lungs, without being obstructed by the scapulae. Considering that the length of the needle used in this case was 70 mm, such needles inserted too deeply into the body by untrained personnel during acupuncture treatment are likely to pierce the pleura and lungs. No obvious injury was found in lungs or pleural after careful examination in the present case. It is possible that the needles used in this case were too fine and the victim's lungs collapsed greatly such that the injuries were not visible. Furthermore, the acupuncture was performed more than 30 hours before death, and the corpse was frozen for several days and thawed before examination. Petechiae in the parietal and visceral pleura may feasibly be absorbed over time, or even vanish during body putrefaction, freezing and thawing of the corpse.

Cases concerning acupuncture-related pneumothorax have been reported.^[1,2,5–7,12–14]^ The majority of the reported victims suffered from unilateral pneumothorax; however, there were also reports of bilateral pneumothorax;^[12–14]^ the pneumothorax was related to needle insertion in the upper back or paraspinal area, similar to that in the present case. In these cases, the time interval between the acupuncture treatment and the appearance of symptoms of pneumothorax ranged from less than an hour, to 2 days. According to those reports, patients with existing respiratory disease, those receiving long-term acupuncture treatment, and those with multiple needle insertion were likely to have presented with rapid respiratory failure shortly after treatment. Moreover, the majority of the patients were not in a critical condition, and the cases of pneumothorax were diagnosed and cured clinically. In the present case, the cause of death was determined to be acute respiratory and circulatory failure due to cardiopulmonary compression and restricted respiration, caused by bilateral tension pneumothorax. No other possible lung diseases that may lead to bilateral spontaneous pneumothorax were found. The needles used in the case were too fine thus leaving tiny perforations in the victim's lungs. A small amount of air continued to slowly enter the chest cavities over a long period. The victim possibly tolerated the mild discomfort and did not pay attention when early symptoms appeared. It took 30 hours to develop into symptoms of severe pneumothorax, and then the victim was sent to the hospital.

In recent years, PMCT has increasingly been applied to forensic examination, and pneumothorax was detected by postmortem imaging in some cases. During conventional autopsy, the pneumothorax test is a special examination process in which the examiner punctures the intercostal muscles under water and observes whether there are bubbles formed. Therefore, it is not part of the routine examination in each autopsy, and forensic pathologists may neglect the process and miss the findings. There also have been case reports regarding the use of PMCT to detect pneumothorax.^[15–18]^ Most of the reported victims suffered from gunshot, or stab wounds, or traffic accidents. Obvious chest injuries other than pneumothorax were detected by both PMCT and body examination. Reports on the use of PMCT to diagnose pneumothorax caused by acupuncture are rare. In this case, there was no obvious chest injury, and the victim could hardly speak when admitted, due to breathing difficulties. In fact, the important information that the victim had recently received acupuncture would have been omitted had there not been careful police investigation. Tension pneumothorax caused by acupuncture is rare and most likely not recognized by forensic pathologists. Total lung collapse and mediastinum shift to the intact side after exposure of the chest cavities may be detected during conventional autopsy, but not every pneumothorax has such significant lung collapse. In the scenario wherein gas cannot be observed in the pleural cavity, direct evidence of pneumothorax will be missed. PMCT is a sensitive tool in detecting gas and can be used to overcome the difficulty mentioned above. In this case, the information obtained from PMCT before the autopsy was used to detect and accurately evaluate the severity of pneumothorax; this information could be used by the forensic pathologists to relate the pinpricks on the back to pneumothorax and remind them of the possibility of acupuncture-related pneumothorax. It could also help to make an effective procedure for the following autopsy. PMCT also has some limitations. PMCT is unable to reveal the true color of the human tissues. And because the resolution of the CT images is closely related to the performance of the machine so minor injuries and lesions may be missed in some cases. In the present case, the information obtained by PMCT was sufficient for the diagnosis of pneumothorax. And the determination of the cause of death was not affected by such limitations. In conclusion, PMCT has shown diagnostic value in tension pneumothorax and played a supporting role in determining the cause of death.

## Author contributions

**Conceptualization:** Jianhua Zhang.

**Data curation:** Lei Wan, Min Zhang.

**Investigation:** Ningguo Liu.

**Project administration:** Yijiu Chen.

**Writing – original draft:** Junqi Jian, Yu Shao.
